# Immunometabolic Plasticity in Sarcopenic Obesity: Toward a New Paradigm for Precision Immunonutrition

**DOI:** 10.3390/nu18172787

**Published:** 2026-08-26

**Authors:** Lucia Malaguarnera

**Affiliations:** Section of Pathology, Department of Biomedical and Biotechnological Sciences, School of Medicine, University of Catania, 95123 Catania, Italy; lucmal@unict.it

**Keywords:** sarcopenic obesity, immunometabolic plasticity, precision immunonutrition, systems biology, adaptive nutrition, bioactive compounds, whole-food matrices, immunometabolism

## Abstract

Immunonutrition continues to generate heterogeneous and often contradictory clinical outcomes, suggesting that nutrients do not exert fixed immunological effects but interact with the biological context in which they operate. Sarcopenic obesity (SO) represents a paradigmatic clinical model of this complexity, where chronic low-grade inflammation, mitochondrial dysfunction, anabolic resistance, metabolic inflexibility, and microbiota remodeling converge to compromise the adaptive capacity of integrated immunometabolic networks. We propose that this condition may be interpreted as a state of impaired immunometabolic plasticity, which may help explain the context-dependent variability of nutritional responses. Within this perspective, micronutrients are viewed not simply as cofactors supporting immune competence but as dynamic regulators of interconnected immunometabolic pathways. Particular attention is devoted to vitamin D and resveratrol, presented as complementary regulators of immunometabolic plasticity. Within the proposed framework, vitamin D may contribute to immunometabolic competence, whereas resveratrol may act as a broader signaling modulator through the SIRT1/AMPK–PGC-1α axis, influencing mitochondrial function, inflammatory tone, metabolic flexibility, and epigenetic adaptation. Beyond isolated compounds, bioactive-rich food matrices, exemplified by *Opuntia ficus-indica*, are discussed as examples of systems-level modulators capable of coordinating inflammatory, metabolic, redox, and microbiota-dependent biological circuitry. This review introduces immunometabolic plasticity as a conceptual framework linking nutritional signals to the coordinated regulation of immune and metabolic adaptation across diverse biological contexts. Finally, we discuss how biomarker-guided phenotyping, multi-omics integration, and context-aware nutritional interventions may provide a foundation for precision immunonutrition, shifting the field from generalized supplementation strategies toward restoration of adaptive immunometabolic resilience.

## 1. Introduction

Despite remarkable advances in nutritional immunology, the clinical efficacy of immunonutrition remains highly variable across chronic metabolic diseases. Nutritional interventions that demonstrate clear biological activity under experimental conditions frequently produce heterogeneous, or even contradictory, clinical outcomes. This variability suggests that nutrients do not exert fixed immunological effects but rather interact with the biological context in which they operate [1]. Increasing evidence indicates that individual responses are influenced not only by nutrient availability but also by the complex physiological state of the host, including immune status, metabolic regulation, mitochondrial function, microbiota composition, and environmental exposures [2]. These observations challenge the traditional reductionist view of immunonutrition, which has largely focused on correcting nutrient deficiencies or targeting isolated molecular pathways, and instead support a more integrated systems-level perspective [3].

Among chronic metabolic disorders, sarcopenic obesity (SO) represents one of the most informative clinical models in which to investigate this biological complexity [4]. Characterized by the coexistence of excess adiposity and progressive skeletal muscle dysfunction, SO is associated with chronic low-grade inflammation, insulin resistance, mitochondrial dysfunction, oxidative stress, anabolic resistance, endocrine alterations, immunosenescence, and gut microbiota remodeling [5]. These processes do not develop independently but interact through highly interconnected regulatory networks that ultimately determine tissue function and clinical outcome [6]. Importantly, patients with apparently similar clinical characteristics often exhibit markedly different responses to nutritional interventions, suggesting that therapeutic efficacy depends on the overall organization and adaptive capacity of these interconnected biological systems rather than on isolated molecular abnormalities [7].

At present, direct mechanistic evidence linking nutritional interventions to these integrated regulatory processes in SO remains limited [8]. Although the biological rationale for immunometabolic interventions is supported by evidence from obesity, aging, metabolic syndrome, type 2 diabetes, and experimental animal models, studies specifically applying standardized diagnostic criteria and biologically characterized SO populations remain scarce [9].

Nevertheless, these complementary lines of evidence indicate that immune regulation, metabolic signaling, mitochondrial homeostasis, redox control, microbial–host interactions, and epigenetic mechanisms are functionally interconnected and may jointly contribute to the maintenance, or disruption, of physiological adaptation. Although not constituting definitive proof, this body of evidence provides a biological rationale for adopting a systems-oriented conceptual framework [5].

Within this perspective, we propose immunometabolic plasticity as a conceptual framework describing the capacity of interconnected immune and metabolic systems to dynamically adapt to nutritional, inflammatory, microbial, endocrine, and environmental stimuli while preserving tissue homeostasis and functional resilience. This concept should not be interpreted as a validated biological entity but as an integrative systems-level model that synthesizes convergent evidence across multiple disciplines and generates experimentally testable hypotheses.

We hypothesize that progressive impairment of immunometabolic plasticity represents a central biological feature of SO and may help explain the heterogeneous responses observed following nutritional interventions. Accordingly, this review examines the molecular mechanisms that may contribute to impaired immunometabolic plasticity, with particular emphasis on inflammatory regulation, nutrient sensing, mitochondrial function, redox homeostasis, and adaptive biological memory. We further discuss how vitamin D, resveratrol, and bioactive-rich whole-food matrices may influence these interconnected regulatory networks and explore how biomarker-guided phenotyping and systems biology approaches could support the future development of precision immunonutrition strategies. Our objective is not to propose new therapeutic recommendations but to provide an integrative framework that can guide future experimental research and facilitate the transition from reductionist nutritional supplementation to the context-aware restoration of adaptive immunometabolic function.

## 2. Biological Basis of Sarcopenic Obesity

Building upon the concepts introduced above, SO is a clinically relevant condition in which chronic metabolic stress progressively reshapes tissue function across multiple organs [10]. Although defined by the coexistence of excess adiposity and impaired skeletal muscle mass and function, SO extends far beyond alterations in body composition [11]. Increasing evidence indicates that SO is characterized by alterations in energy metabolism, endocrine signaling, tissue remodeling, and chronic low-grade inflammation, ultimately compromising the capacity to maintain physiological homeostasis [10].

A central feature of SO is the progressive remodeling of adipose tissue. As adiposity increases, adipocytes undergo hypertrophy and functional alterations that favor local hypoxia, immune cell recruitment, extracellular matrix remodeling, and changes in adipokine secretion [6]. These events transform adipose tissue from an efficient energy storage compartment into a metabolically active organ capable of influencing distant tissues through endocrine, paracrine, and immune-mediated signals [12]. Together, chronic low-grade inflammation, ectopic lipid deposition, and altered metabolic signaling interact to establish a biological environment that progressively favors tissue dysfunction over adaptive remodeling [5]. Recent evidence identifies hypertrophic adipocytes as active orchestrators of immunometabolic remodeling during obesity. Beyond their traditional role as energy storage cells, adipocytes integrate metabolic stress signals and coordinate inflammatory communication with resident immune cells, thereby shaping the progression of chronic low-grade inflammation and systemic metabolic dysfunction [13].

Skeletal muscle undergoes parallel remodeling that extends beyond the quantitative loss of muscle mass [14]. Chronic exposure to inflammatory mediators, lipid overload, and metabolic stress impairs muscle protein turnover, mitochondrial function, regenerative capacity, and contractile performance [15]. Experimental studies further indicate that defective muscle repair is accompanied by alterations in fibro-adipogenic progenitor cell behavior, promoting intramuscular adipose infiltration and progressive deterioration of muscle quality [16]. Consequently, muscle dysfunction in SO reflects an active pathological remodeling process involving mechanisms that cannot be fully explained by aging or physical inactivity alone [14].

The reciprocal interaction between adipose tissue and skeletal muscle further amplifies disease progression [15]. Excess lipid availability, altered adipokine production, impaired myokine signaling, and persistent inflammatory activation establish a self-perpetuating cycle in which dysfunction in one tissue accelerates pathological changes in the other [13]. This bidirectional communication amplifies metabolic dysfunction and progressively impairs tissue function, contributing to the clinical evolution of SO.

**Figure 1:**
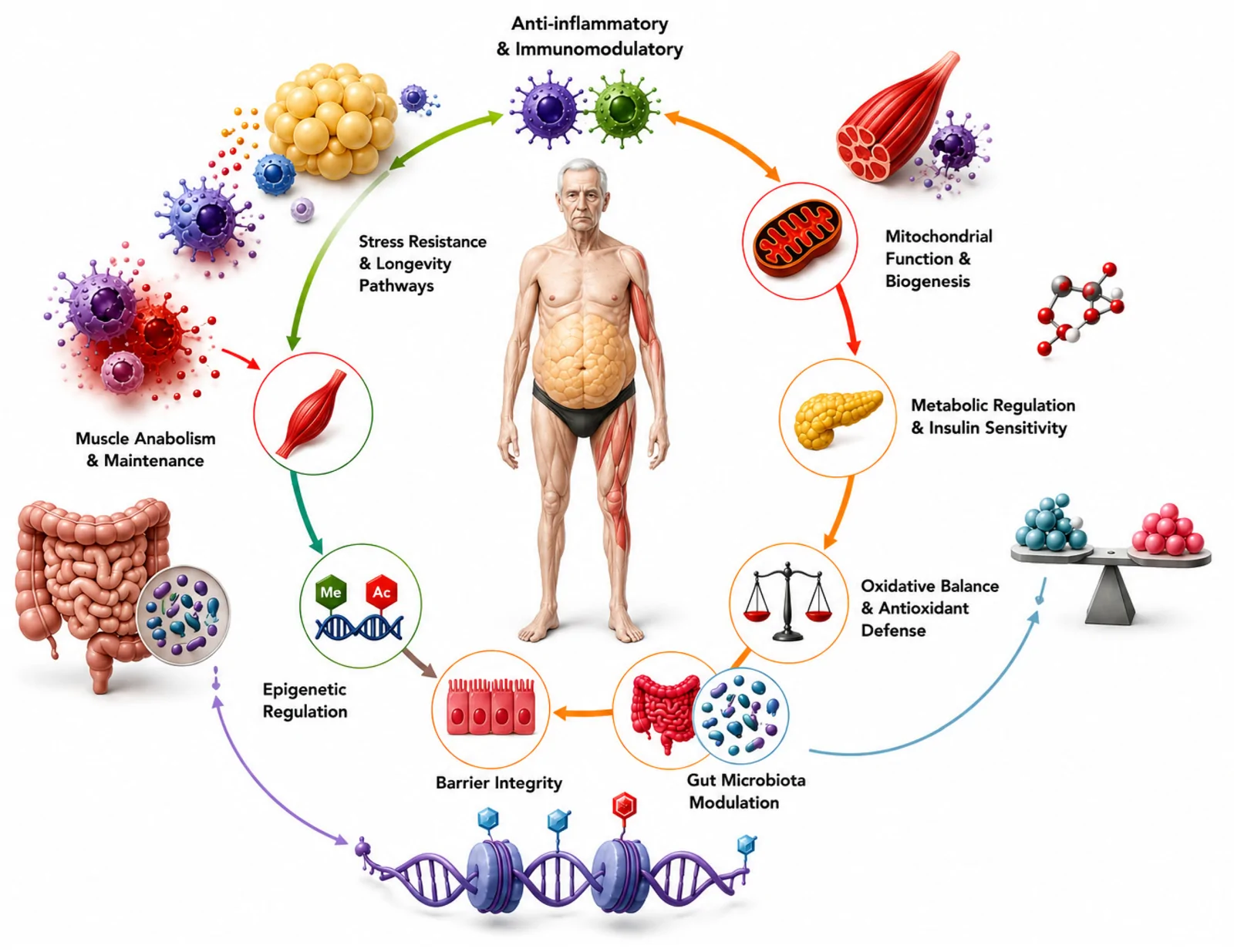
Biological remodeling underlying sarcopenic obesity. Progressive alterations in adipose tissue, skeletal muscle, immune function, mitochondrial homeostasis, gut microbiota, epigenetic regulation, and systemic metabolic homeostasis interact within an interconnected network associated with tissue dysfunction in sarcopenic obesity. Continuous crosstalk among these biological compartments may contribute to disease progression and to the marked biological heterogeneity of sarcopenic obesity. Arrows represent reciprocal communication among biological compartments and should be interpreted as dynamic bidirectional interactions rather than linear causal pathways.

An additional hallmark of SO is its marked biological heterogeneity. Individuals with apparently similar anthropometric characteristics differ in the degree of adipose tissue dysfunction, muscle quality, mitochondrial competence, endocrine regulation, and inflammatory activation [17]. Likewise, responses to nutritional interventions remain highly variable, suggesting that conventional phenotypic classification captures only part of the underlying biological complexity [18]. Emerging evidence on circulating mediators involved in adipose–muscle communication further suggests the existence of distinct biological phenotypes that may influence disease progression and therapeutic responsiveness [15]. Collectively, these observations indicate that SO is a biologically heterogeneous syndrome characterized by coordinated remodeling of adipose tissue, skeletal muscle, and their reciprocal communication. Understanding the molecular mechanisms underlying these biological alterations is therefore essential for explaining disease progression and the heterogeneous responses to nutritional interventions (Figure 1).

## 3. Molecular Pathways Underlying Impaired Immunometabolic Plasticity in Sarcopenic Obesity

The biological remodeling described in the previous section involves a coordinated set of molecular pathways that regulate inflammatory signaling, nutrient sensing, mitochondrial function, redox homeostasis, and cellular adaptation.

For clarity, these pathways are discussed as distinct functional modules, although extensive crosstalk exists among them.

### 3.1. NF-κB and Nrf2: The Balance Between Inflammation and Adaptive Resilience

A central event contributing to impaired immunometabolic plasticity in SO is the progressive disruption of the physiological balance between inflammatory activation and antioxidant defense. Chronic low-grade inflammation associated with excess adiposity promotes persistent activation of nuclear factor kappa B (NF-κB), leading to sustained production of pro-inflammatory cytokines, immune cell activation, and increased oxidative stress [19]. Under physiological conditions, NF-κB plays an essential role in host defense and tissue homeostasis. However, persistent activation progressively compromises muscle homeostasis, insulin sensitivity, and mitochondrial function [5,20].

Experimental and clinical studies indicate that sustained NF-κB activation in obesity and metabolic dysfunction is associated with persistent inflammatory signaling, impaired insulin sensitivity, mitochondrial dysfunction, and activation of muscle atrophy pathways [21,22,23]. Collectively, these findings support the involvement of NF-κB in biological processes relevant to SO.

In contrast, nuclear factor erythroid 2-related factor 2 (Nrf2) orchestrates antioxidant defense, mitochondrial protection, detoxification pathways, and cellular adaptation to oxidative stress [24,25]. In healthy tissues, reciprocal regulation between NF-κB and Nrf2 limits excessive inflammatory activation while preserving redox homeostasis and tissue resilience [26]. In SO, however, this regulatory balance is progressively disrupted, favoring persistent inflammatory signaling while weakening antioxidant and cytoprotective responses. The resulting redox imbalance amplifies mitochondrial dysfunction, anabolic resistance, and impaired tissue repair, potentially contributing to the progressive loss of immunometabolic plasticity [27].

### 3.2. AMPK–mTOR–FOXO–SIRT1: Nutrient Sensing and Metabolic Adaptation

Maintenance of skeletal muscle mass and metabolic flexibility depends on the coordinated activity of nutrient-sensing pathways integrating cellular energy status with immune regulation, mitochondrial function and protein turnover. Among these, AMP-activated protein kinase (AMPK), the mechanistic target of rapamycin (mTOR), Forkhead box O transcription factors (FOXO), and sirtuin 1 (SIRT1) constitute a central regulatory axis governing immunometabolic adaptation [28].

AMPK serves as a principal cellular energy sensor, promoting oxidative metabolism, mitochondrial biogenesis, autophagy, and metabolic flexibility under conditions of energetic stress [29]. In contrast, mTOR coordinates anabolic metabolism, protein synthesis, skeletal muscle maintenance, and immune cell proliferation in response to nutrient availability. FOXO transcription factors regulate stress resistance, autophagy, antioxidant defense, and muscle protein turnover, whereas SIRT1 integrates nutrient availability with mitochondrial quality control, metabolic adaptation, and epigenetic remodeling [30].

Evidence from obesity, aging, and experimental models of metabolic dysfunction indicates that alterations in AMPK, mTOR, FOXO, and SIRT1 signaling are associated with mitochondrial dysfunction, anabolic resistance, and progressive skeletal muscle deterioration, highlighting the interconnected nature of nutrient-sensing regulation [31]. A defining feature of SO is the coexistence of chronic nutrient excess with progressively impaired nutrient sensing. Chronic metabolic overload, insulin resistance, mitochondrial dysfunction, and persistent inflammatory signaling progressively uncouple these interconnected pathways, impairing their ability to coordinate cellular adaptation. Collectively, disruption of the AMPK–mTOR–FOXO–SIRT1 regulatory axis compromises metabolic adaptability and may contribute to the progressive loss of immunometabolic plasticity in SO [32].

### 3.3. Network Failure: From Adaptive Plasticity to Chronic Dysfunction

The molecular pathways described above do not function as isolated signaling cascades but as components of an integrated regulatory network that continuously coordinates immune responses, cellular metabolism, and tissue adaptation.

Dynamic communication among inflammatory signaling, nutrient sensing mechanisms, mitochondrial quality control, redox homeostasis, endocrine mediators, and microbiota-derived signals enables organisms to respond efficiently to metabolic and environmental challenges while preserving physiological homeostasis.

**Figure 2:**
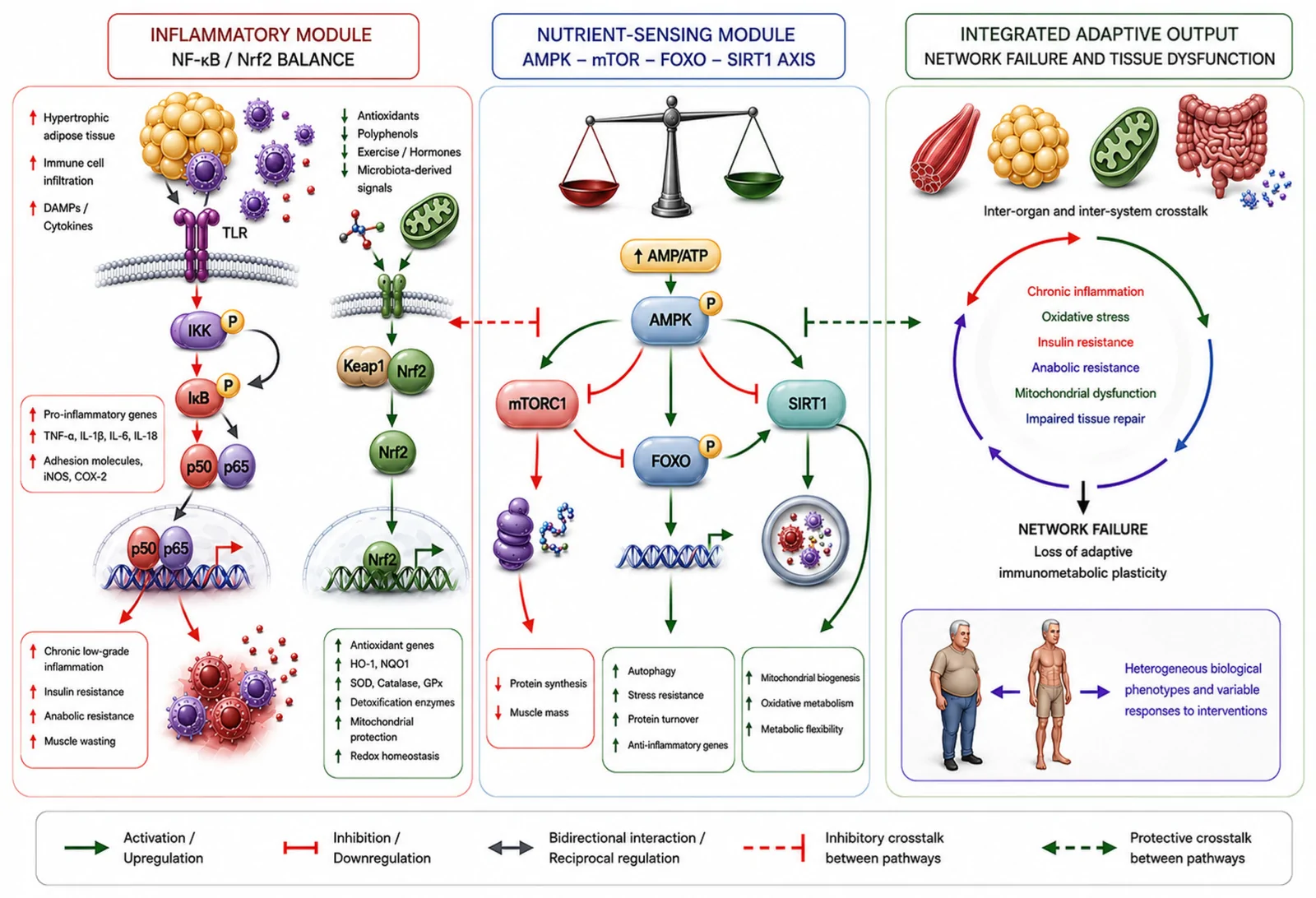
Coordinated molecular pathways underlying impaired immunometabolic plasticity in sarcopenic obesity. Chronic metabolic stress promotes persistent activation of the TLR–IKK–NF-κB pathway, sustaining inflammatory signaling, insulin resistance, anabolic resistance, and muscle wasting. In parallel, dysregulation of the Keap1–Nrf2 axis may weaken antioxidant defenses, mitochondrial protection, and redox homeostasis. The nutrient-sensing network composed of AMPK, mTOR, FOXO, and SIRT1 integrates cellular energy status with protein synthesis, autophagy, mitochondrial biogenesis, and metabolic adaptation. Progressive dysregulation of these interconnected pathways may contribute to chronic inflammation, mitochondrial dysfunction, impaired tissue repair, and progressive loss of adaptive immunometabolic plasticity in sarcopenic obesity. Abbreviations: AMPK, AMP-activated protein kinase; FOXO, Forkhead box O transcription factors; GPx, glutathione peroxidase; HO-1, heme oxygenase-1; IκB, inhibitor of nuclear factor kappa B; IKK, IκB kinase; Keap1, Kelch-like ECH-associated protein 1; mTOR, mechanistic target of rapamycin; NF-κB, nuclear factor kappa B; NQO1, NAD(P)H quinone oxidoreductase 1; Nrf2, nuclear factor erythroid 2-related factor 2; SIRT1, sirtuin 1; SOD, superoxide dismutase; TLR, Toll-like receptor.

In SO, progressive dysregulation of these interconnected pathways extends beyond individual cellular dysfunctions, compromising the coordinated integration of biological responses across multiple tissues and organs. The resulting loss of coordination may amplify chronic inflammation, metabolic dysfunction, anabolic resistance, and impaired tissue regeneration, thereby favoring the transition from adaptive regulation to persistent pathological remodeling [33]. This systems-level organization provides the mechanistic rationale for considering nutritional strategies capable of modulating multiple interconnected regulatory pathways. Within the proposed framework, such an integrated approach may offer greater potential to support adaptive immunometabolic function than interventions directed toward a single molecular target, although this hypothesis requires experimental and clinical validation (Figure 2).

## 4. Vitamin D and Resveratrol: Complementary Regulators of Immunometabolic Plasticity

The systems-level perspective described above suggests that restoring immunometabolic plasticity is unlikely to depend on the suppression of a single pathological process. Instead, recovery of adaptive function may require coordinated modulation of the interconnected regulatory networks governing immune responses, cellular metabolism, mitochondrial homeostasis, and tissue resilience [34]. Within this context, dietary bioactive molecules represent an additional layer of biological regulation that extends beyond their classical nutritional functions.

**Figure 3:**
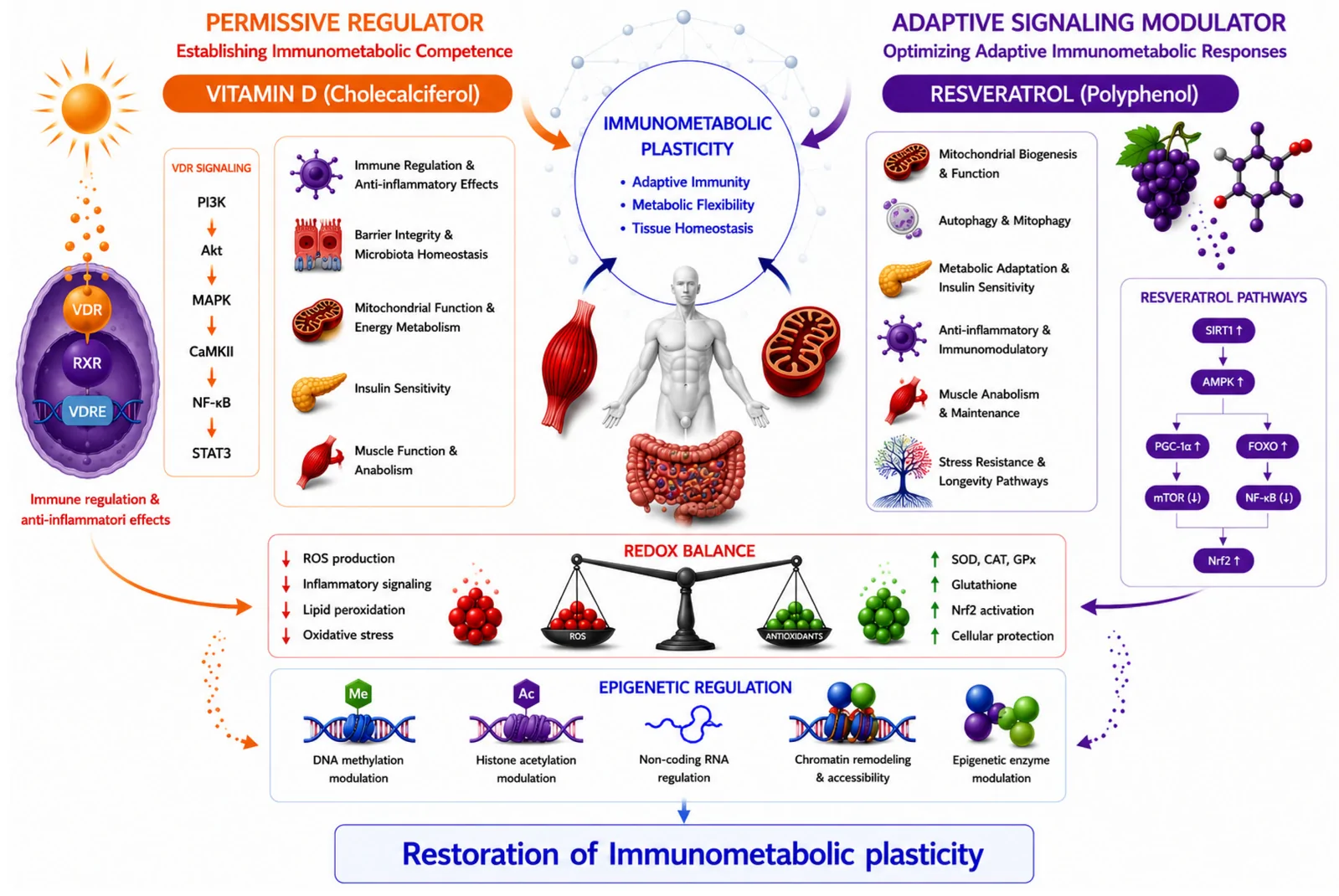
Complementary regulatory roles of vitamin D and resveratrol in immunometabolic plasticity. Vitamin D may support immunometabolic competence through VDR-mediated regulation of immune homeostasis, mitochondrial activity, barrier integrity, metabolic function, and gene transcription. Resveratrol modulates interconnected signaling pathways involving SIRT1, AMPK, PGC-1α, mTOR, and FOXO, with reported effects on mitochondrial biogenesis, metabolic adaptation, autophagy, cellular stress responses, inflammatory regulation, and antioxidant defenses. Both compounds also influence redox homeostasis and epigenetic regulatory mechanisms, illustrating complementary dimensions of biological regulation that, within the proposed framework, may contribute to immunometabolic plasticity. Abbreviations: AMPK, AMP-activated protein kinase; FOXO, Forkhead box O transcription factors; mTOR, mechanistic target of rapamycin; NF-κB, nuclear factor kappa B; Nrf2, nuclear factor erythroid 2-related factor 2; PGC-1α, peroxisome proliferator-activated receptor gamma coactivator 1-alpha; SIRT1, sirtuin 1; VDR, vitamin D receptor.

From this perspective, vitamin D and resveratrol can be viewed as complementary examples of systems-level immunometabolic modulators. Although they act through distinct molecular mechanisms, both influence multiple interconnected pathways involved in immune regulation, mitochondrial function, metabolic adaptation, and tissue homeostasis [8,35] (Figure 3).

### 4.1. Vitamin D: Supporting Immunometabolic Competence

Vitamin D represents one of the most extensively studied micronutrients involved in the regulation of immune and metabolic homeostasis. Beyond its classical role in calcium and bone metabolism, vitamin D exerts pleiotropic effects through activation of the vitamin D receptor (VDR), which is expressed in numerous immune, metabolic, and musculoskeletal tissues. VDR signaling influences innate and adaptive immunity, inflammatory regulation, skeletal muscle function, insulin sensitivity, mitochondrial activity, and epithelial barrier integrity, while also contributing to the maintenance of gut microbiota homeostasis [36].

Within the framework of immunometabolic plasticity, vitamin D may therefore be regarded as a permissive regulator that establishes the biological conditions required for coordinated adaptive responses.

By supporting the functional competence of multiple interconnected systems, adequate vitamin D status may facilitate the ability of immune and metabolic networks to respond appropriately to nutritional and environmental challenges [37].

Although vitamin D supplementation alone may not fully restore impaired immunometabolic plasticity in biologically complex disorders such as SO, insufficient vitamin D availability may compromise the capacity of these adaptive networks to function efficiently [38]. Consequently, adequate vitamin D status may represent an important biological condition supporting immunometabolic resilience in the context of future precision immunonutrition strategies [8] (Figure 3).

### 4.2. Resveratrol: Modulating Adaptive Immunometabolic Responses

Among signaling bioactive compounds, resveratrol represents one of the most compelling examples of a systems-level regulator of immunometabolic plasticity. Unlike classical antioxidants or anti-inflammatory agents, resveratrol does not primarily exert its biological effects through direct inhibition of individual signaling pathways. Instead, it enhances the adaptive capacity of interconnected immune and metabolic networks—enabling them to respond more effectively to persistent biological stress [39].

This systems-level activity is mediated by coordinated modulation of multiple adaptive pathways involved in mitochondrial quality control, nutrient sensing, oxidative metabolism, inflammatory resolution, and cellular stress responses. Activation of SIRT1, together with regulation of the AMPK-peroxisome proliferator-activated receptor gamma coactivator 1-alpha (PGC-1α), mTOR, and FOXO signaling axis, promotes mitochondrial biogenesis, improves oxidative phosphorylation, stimulates autophagy, maintains anabolic–catabolic balance, and reinforces cellular resilience under conditions of metabolic stress. Simultaneously, resveratrol attenuates chronic inflammatory activation through indirect regulation of NF-κB while facilitating antioxidant responses coordinated by Nrf2, thereby contributing to restoration of redox homeostasis [8,35] (Figure 3).

Within the context of SO, these coordinated actions may partially counteract the progressive loss of adaptive flexibility that characterizes the disease. By modulating multiple interconnected regulatory circuits, resveratrol may contribute to the functional reorganization of immunometabolic networks. Consequently, its biological effects can be interpreted as emerging from coordinated network regulation rather than pathway-specific inhibition.

From the perspective of precision immunonutrition, resveratrol may therefore be viewed as an adaptive signaling modulator whose actions complement those of essential micronutrients. Whereas vitamin D may support the biological competence required for coordinated immune and metabolic function, resveratrol may modulate signaling pathways involved in the efficiency and adaptability of these interconnected systems during chronic metabolic stress. Together, they illustrate how distinct nutritional components may influence complementary dimensions of immunometabolic regulation.

## 5. Whole-Food Matrices as Systems-Level Modulators of Immunometabolic Plasticity in Sarcopenic Obesity

Whole-food matrices mirror the intrinsic complexity of human physiology. Unlike isolated micronutrients or purified bioactive compounds, naturally occurring food matrices contain highly organized combinations of vitamins, minerals, polyphenols, dietary fibers, lipids, and numerous phytochemicals that interact dynamically during digestion, microbial metabolism, cellular uptake, and intracellular signaling [40]. Their biological activity may therefore reflect coordinated interactions among multiple constituents, extending beyond the effects attributable to individual molecules.

Within the context of SO, these interactions are particularly relevant because they may simultaneously influence several mechanisms associated with impaired immunometabolic plasticity. Bioactive food matrices influence inflammatory signaling, mitochondrial quality control, oxidative stress, nutrient sensing, gut microbiota composition, endocrine communication, and skeletal muscle metabolism through integrated biological responses involving multiple interconnected pathways [41]. This multidimensional activity provides a biological rationale for considering whole-food matrices as potential modulators of adaptive immunometabolic responses.

**Figure 4:**
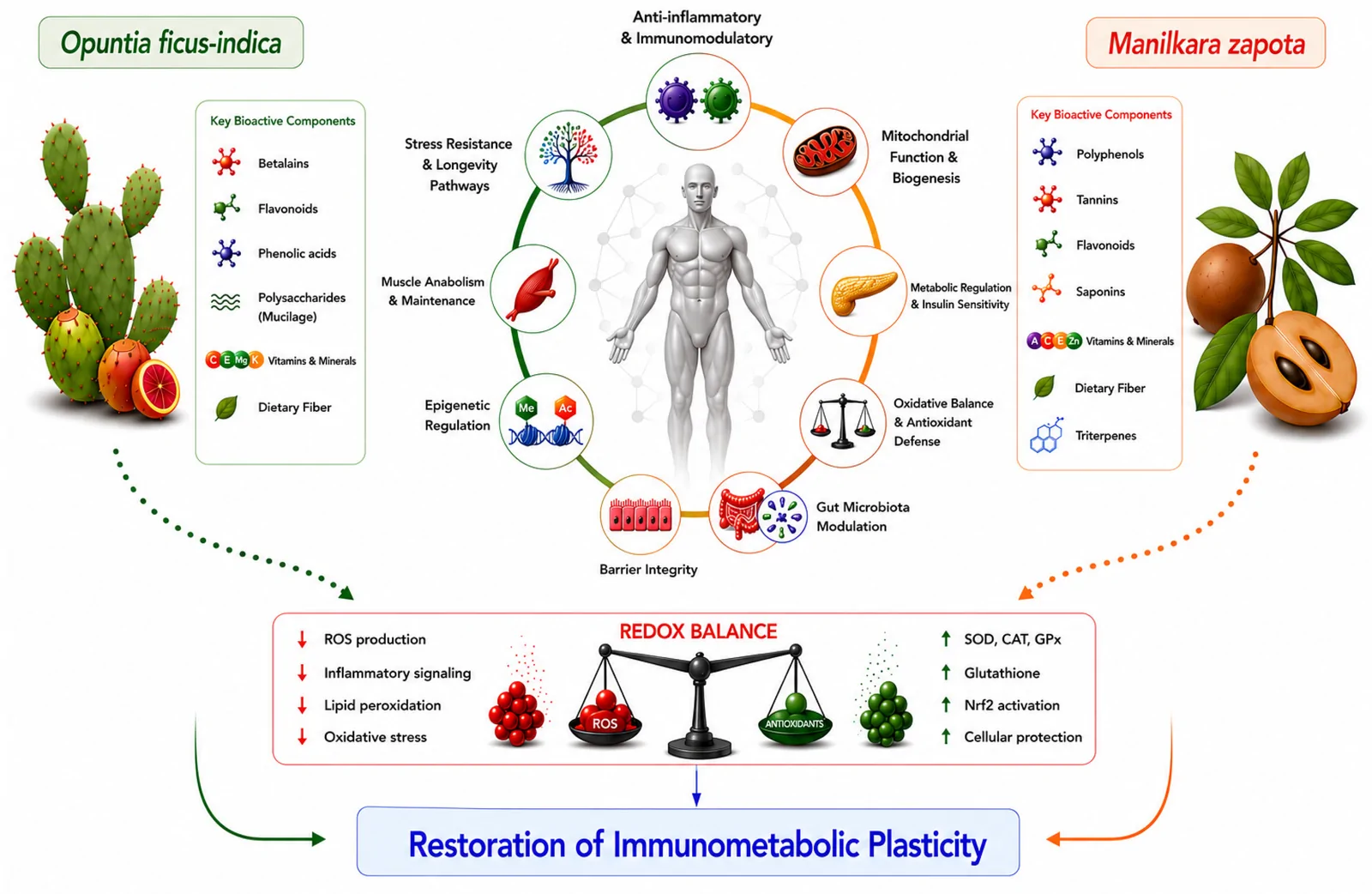
Mechanistic integration of whole-food matrix-derived signals in immunometabolic regulation: *Opuntia ficus-indica* and *Manilkara zapota* as complementary models. The complex phytochemical and nutritional profiles of *Opuntia ficus-indica* and *Manilkara zapota*, including polyphenols, flavonoids, betalains, tannins, saponins, dietary fiber, vitamins, and minerals, may engage multiple interconnected biological pathways. Their reported actions include modulation of inflammatory and immune signaling, mitochondrial function and biogenesis, glucose and insulin-related metabolic regulation, skeletal muscle homeostasis, epithelial barrier integrity, gut microbiota composition, and epigenetic regulation. These effects converge on redox control through reduced reactive oxygen species generation and lipid peroxidation, together with reinforcement of endogenous antioxidant defenses involving Nrf2, glutathione, superoxide dismutase, catalase, and glutathione peroxidase. Through the coordinated influence of these molecular and cellular processes, whole-food matrices may support adaptive immunometabolic responses and provide mechanistic models for investigating nutrition-driven modulation of immunometabolic plasticity. Abbreviations: CAT, catalase; GPx, glutathione peroxidase; Nrf2, nuclear factor erythroid 2-related factor 2; ROS, reactive oxygen species; SOD, superoxide dismutase.

Among naturally occurring food matrices, *Opuntia ficus-indica* and *Manilkara zapota* represent useful translational models illustrating how biologically organized combinations of micronutrients and phytochemicals regulate complex adaptive systems [42,43]. Although these fruits are geographically restricted and are not intended as universal nutritional recommendations, they illustrate how biological activity may depend not only on chemical composition but also on interactions among their bioactive constituents. Their reported effects on inflammation, oxidative stress, mitochondrial function, gut microbiota, and epigenetic regulation provide examples of multidimensional biological activity that, within the proposed framework, may be relevant to immunometabolic plasticity (Figure 4).

From a conceptual perspective, these natural matrices may therefore be viewed not simply as potential functional foods but as biological prototypes illustrating how complex combinations of dietary constituents can influence interconnected immune–metabolic responses. Understanding these principles provides opportunities to translate naturally occurring biological complexity into more accessible nutritional interventions.

From a translational perspective, the objective would not be to reproduce specific fruits, but to identify organizational principles through which combinations of micronutrients, phytochemicals, fibers, and other bioactive constituents generate coordinated biological responses. Characterizing these interactions could inform the future development of standardized nutraceutical formulations, bioactive-enriched functional foods, and engineered food matrices designed to reproduce selected properties of natural matrices while improving accessibility, reproducibility, and clinical applicability [40].

Whole-food matrices may therefore provide useful models for investigating how the integrated activity of multiple bioactive constituents influences coordinated immunometabolic function [44].

## 6. Adaptive Memory as a Component of Immunometabolic Plasticity in Sarcopenic Obesity

One of the defining characteristics of biological plasticity is its ability to retain information from previous environmental exposures. Nutritional signals not only induce transient metabolic responses but can also generate persistent molecular adaptations that influence how immune and metabolic systems respond to future challenges [45]. This phenomenon may be conceptualized as adaptive memory, whereby previous nutritional and environmental experiences shape subsequent biological responsiveness [46]. While such persistent adaptations may support biological resilience under physiological conditions, chronic metabolic and inflammatory stress in SO may favor the stabilization of maladaptive programs that perpetuate immune and metabolic dysfunction [47].

The molecular basis of adaptive memory may involve coordinated interactions among epigenetic regulation, mitochondrial metabolism, and redox signaling [48].

These mechanisms operate as interconnected regulatory circuits that translate repeated environmental and nutritional stimuli into long-lasting modifications of cellular phenotype and function.

Epigenetic regulation represents one of the principal mechanisms through which nutritional information becomes biologically embedded. Micronutrients involved in one-carbon metabolism, including folate, vitamins B12 and B6, and choline, regulate methyl-group availability required for DNA and histone methylation, whereas vitamin C, zinc, selenium, and other redox-active micronutrients influence chromatin remodeling through modulation of oxidative balance and enzyme activity [49]. Collectively, nutritional status continuously shapes gene expression without altering DNA sequence [50]. Vitamin D and resveratrol may influence molecular processes relevant to adaptive memory through epigenetic and transcriptional mechanisms. Vitamin D modulates VDR-dependent transcriptional programs [51], whereas resveratrol influences epigenetic and mitochondrial regulatory pathways [39]. Within the proposed framework, these actions may contribute to persistent biological adaptations extending beyond immediate metabolic and immunological responses (Figure 3). A further component of adaptive memory derives from redox regulation. Reactive oxygen species are now recognized not only as metabolic by-products but also as signaling mediators controlling transcription factors, mitochondrial adaptation, inflammatory responses, and epigenetic enzymes [52]. These mechanisms converge with the emerging concept of trained immunity, whereby metabolic and epigenetic reprogramming induces durable functional changes in innate immune cells. Together, these processes provide a mechanistic basis for considering epigenetic regulation, redox signaling, mitochondrial adaptation, and trained immunity as interconnected components of adaptive memory that may influence long-term immunometabolic responsiveness [53].

From this perspective, nutritional interventions may influence not only current immune and metabolic function but also the biological processes through which previous nutritional and inflammatory exposures shape future responsiveness. Consequently, modulation of adaptive memory may represent one component of strategies aimed at restoring immunometabolic plasticity, although its relevance to nutritional responsiveness in SO requires direct experimental validation.

## 7. Precision Immunonutrition: Translating Immunometabolic Plasticity into Clinical Practice

Viewing SO through the framework of immunometabolic plasticity broadens the objectives of nutritional intervention. Conventional nutritional approaches have primarily focused on correcting nutrient deficiencies or administering isolated bioactive compounds. However, the marked biological heterogeneity observed among individuals with SO suggests that nutritional efficacy depends not only on nutrient availability but also on the adaptive capacity of each patient’s immunometabolic system [1].

The proposed framework should be interpreted within the broader context of established multimodal management of SO. Adequate energy intake, sufficient dietary protein, appropriate macronutrient composition, and regular resistance exercise remain the cornerstone of current therapeutic strategies aimed at preserving skeletal muscle mass and metabolic health. Within this context, vitamin D, resveratrol, and bioactive-rich whole-food matrices should not be considered stand-alone therapeutic interventions but complementary modulators that may enhance the adaptive capacity of interconnected immunometabolic systems. Their biological effects are therefore likely to depend on the overall nutritional and functional environment in which they operate, including macronutrient availability, energy balance, and anabolic stimuli. Although these interactions are biologically plausible, their magnitude and clinical relevance remain to be established through well-designed prospective studies.

From this perspective, precision immunonutrition may evolve from generalized supplementation toward phenotype-guided nutritional strategies capable of addressing the predominant mechanisms associated with impaired immunometabolic plasticity in individual patients [3]. Such an approach would recognize the biological heterogeneity of individuals with SO and support the future development of more individualized nutritional interventions [1].

Achieving this objective requires integration of multiple biological dimensions. Circulating inflammatory biomarkers, metabolomic and lipidomic profiles, gut microbiota composition, mitochondrial function, epigenetic signatures, body composition analysis, and digital health technologies collectively provide the opportunity to characterize patient-specific immunometabolic phenotypes with increasing precision. Such multidimensional profiling may facilitate the identification of those individuals most likely to benefit from specific combinations of micronutrients, signaling bioactive compounds, and whole-food matrix-based interventions. Operationally, immunometabolic plasticity should be regarded as a dynamic property that cannot be inferred solely from static biomarker concentrations. Future assessment frameworks will likely integrate baseline measurements with responses to standardized nutritional, metabolic, or physical challenges. Candidate domains include inflammatory regulation, glucose and lipid metabolism, mitochondrial function, redox homeostasis, skeletal muscle performance, endocrine signaling, and host-microbiota interactions. Future evaluation of immunometabolic plasticity could integrate the magnitude, timing, coordination, and reversibility of biological responses across multiple functional domains. Within this proposed framework, impaired plasticity would be reflected not by isolated biomarker abnormalities but by maladaptive response patterns that are attenuated, excessive, delayed, poorly coordinated, or incompletely reversible. Conversely, preserved or restored plasticity would be characterized by coordinated adaptation and timely recovery toward physiological homeostasis. Although no validated composite score is currently available, this framework generates experimentally testable hypotheses that may support future biological characterization of SO.

Translating this concept into clinical practice will require a stepwise implementation strategy. At present, comprehensive multi-omics profiling, mitochondrial functional assessment, epigenetic characterization, digital health technologies, and artificial intelligence-based predictive models remain primarily research tools because of their cost, limited standardization, restricted accessibility, and uncertain incremental clinical value in routine care. Consequently, precision immunonutrition should be viewed as a progressive translational framework rather than an immediately applicable clinical model.

A pragmatic implementation strategy may therefore distinguish two complementary levels of assessment. At the research level, advanced multidimensional profiling integrating multi-omics data, systems biology approaches, and longitudinal analyses may enable high-resolution characterization of immunometabolic phenotypes. In routine clinical practice, however, an initial phenotype-guided approach may rely on more readily available clinical, biochemical, and functional measures, including body composition, muscle strength and physical performance, inflammatory biomarkers (e.g., C-reactive protein), fasting glucose, insulin resistance, lipid profile, nutritional assessment, and selected circulating biomarkers when available. Although these variables cannot directly quantify immunometabolic plasticity, they may provide practical surrogate indicators of adaptive immunometabolic function until more comprehensive approaches become clinically feasible. Future studies should determine whether combining these accessible clinical variables with selected molecular biomarkers can improve biological phenotyping, predict responsiveness to nutritional interventions, and monitor restoration of adaptive function over time. Their validity as surrogate measures of immunometabolic plasticity, however, remains to be established through prospective longitudinal studies.

As systems biology, artificial intelligence, and multi-omics technologies continue to evolve, these approaches may further refine precision immunonutrition by enabling predictive models capable of matching nutritional interventions to individual biological profiles. These technologies should complement clinical expertise by supporting more informed nutritional decision-making, with the potential to improve therapeutic efficacy and reduce unnecessary or ineffective interventions.

Ultimately, the goal of precision immunonutrition extends beyond personalized nutrient supplementation toward restoration of adaptive biological function.

The ultimate objective would be not simply to optimize nutrient intake but to support the capacity of interconnected immune and metabolic systems to dynamically adapt to changing physiological demands, with the long-term aim of improving metabolic health, preserving skeletal muscle function, and reducing frailty in individuals with SO.

## 8. Conclusions and Future Perspectives

This review proposes immunometabolic plasticity as a conceptual framework for interpreting the biological complexity of SO. Instead of viewing SO solely as the coexistence of obesity and skeletal muscle loss, this perspective interprets the condition in relation to progressive impairment of the adaptive interactions that normally coordinate immune regulation, metabolism, mitochondrial function, redox homeostasis, endocrine signaling, and host-microbiota communication. Within this systems-level framework, nutritional interventions may be interpreted not solely in terms of the biological activity of individual nutrients or isolated signaling pathways, but also according to their potential to influence coordinated adaptive function across interconnected biological systems. Essential micronutrients, signaling bioactive compounds such as resveratrol, and naturally organized whole-food matrices may therefore influence complementary dimensions of biological regulation relevant to immunometabolic plasticity. Beyond its relevance to SO, the concept of immunometabolic plasticity may also provide a broader conceptual framework for understanding the context-dependent efficacy of nutritional interventions across chronic inflammatory and metabolic disorders. By emphasizing adaptive regulation alongside individual molecular targets, this perspective may help bridge nutritional science, systems biology, immunology, and precision medicine within a unified biological model. Perhaps the most important implication of this paradigm is a shift in how nutrition itself is viewed. Future progress may depend less on identifying additional bioactive molecules than on understanding how biological systems integrate nutrients, phytochemicals, microbiota-derived metabolites, and environmental signals into coordinated adaptive responses. In this context, naturally occurring food matrices may represent useful biological models whose organizational principles could inform the development of next-generation nutraceuticals, functional foods, and engineered nutritional platforms designed to support coordinated biological responses. Ultimately, the future of precision immunonutrition may depend on understanding how nutrition influences coordinated network function within complex immunometabolic systems. If validated through future experimental and clinical studies, immunometabolic plasticity may evolve from a conceptual framework into a practical biological principle for guiding next-generation nutritional strategies in SO and, potentially, other chronic diseases.

## Data Availability

No new data were created or analyzed in this study. Data sharing is not applicable to this article
